# Supplementary material for: Mesenchymal Stem Cell Use in Acute Tendon Injury: In Vitro Tenogenic Potential vs. In Vivo Dose Response
Source: Bioengineering (Basel). 2022 Aug 22;9(8):407. doi: 10.3390/bioengineering9080407 (PMC9404841; doi:10.3390/bioengineering9080407)
Supplement: Supplementary file 1 [file bioengineering-09-00407-s001.zip › bioengineering-1885452-supplementary.pdf]

bioengineering

Article

Mesenchymal Stem Cell Use in Acute Tendon Injury: In Vitro Tenogenic Potential vs. In Vivo Dose Response

Kristin Bowers [1], Lisa Amelse [1] , Austin Bow [1] , Steven Newby [1] , Amber MacDonald [1] , Xiaocun Sun [1] , David Anderson [1] , and Madhu Dhar [1,*]

[1] Large Animal Clinical Sciences, University of Tennessee College of Veterinary Medicine, Knoxville, Tennessee, USA
* Correspondence: kbower17@vols.utk.edu

Abstract: Stem cell therapy for the treatment of tendon injury is an emerging clinical practice in the fields of human and veterinary sports medicine. However, the therapeutic benefit of intralesional transplantation of mesenchymal stem cells in tendonitis cases is not well designed. Questions persist regarding the overall tenogenic potential and efficacy of this treatment alone. In this study, we aimed to isolate a rat mesenchymal stem cell lineage for in vitro and in vivo use, to assess the effects of growth factor exposure in vitro on cell morphology, behavior, and tendon-associated glycoprotein production, and to assess the therapeutic potential of intralesional stem cells, as a function of dose, in vivo. First, rat adipose-derived (rAdMSC) and bone marrow-derived (rBMSC) stem cell lineages were isolated, characterized with flow cytometric analysis, and compared in terms of proliferation (MTS assay) and cellular viability (calcein AM staining). Rat AdMSCs displayed superior proliferation and more homogenous CD 73, CD 44H, and CD 90 expression as compared to rBMSC. Next, the tenogenic differentiation potential of the rAdMSC lineage was tested in vitro through isolated and combined stimulation with reported tenogenic growth factors, transforming growth factor (TGF)-β3 and connective tissue growth factor (CTGF). We found that the most effective tenogenic factor in terms of cellular morphologic change, cell alignment/orientation, sustained cellular viability, and tendon-associated glycoprotein upregulation was TGFβ3, and we confirmed that rAdMSC could be induced toward a tenogenic lineage in vitro. Finally, the therapeutic potential of rAdMSCs as a function of dose was assessed using a rat acute Achilles tendon injury model. 5×10^5 (low dose) and 4×10^6 (high dose) were used. Subjectively, on gross morphology, rAdMSC-treated tendons exhibited fewer adhesions and less scar tissue than control tendons. However, regardless of rAdMSC dose, no significant differences in histological grade or tissue collagen I deposition were noted between rAdMSC-treated and control tendons. Collectively, rAdMSCs exhibited appropriate stem cell markers and tenogenic potential in vitro, but clinical efficacy of intralesional implantation of undifferentiated cells in acute tendonitis cases could not be proven. Further investigation into complementary therapeutics or specialized culture conditions prior to implantation are warranted.

Keywords: mesenchymal stem cell 1; tendon 2; transforming growth factor beta-3 3; connective tissue growth factor 4; extracellular matrix 5; tenogenic differentiation 6

Supplementary

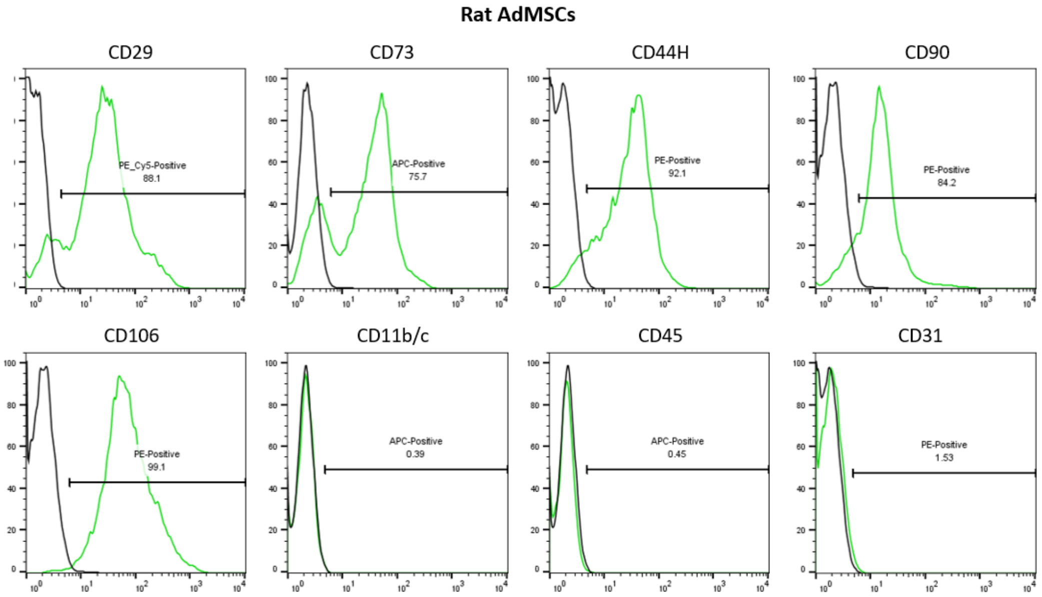

Figure S1. Flow cytometry results for rAdMSC and rBMSC.
